# Canadian Armed Forces Veterans’ Perspectives on the Effects of Exposure to Children in Armed Conflict During Military Service: Protocol for a Qualitative Study

**DOI:** 10.2196/57146

**Published:** 2024-06-14

**Authors:** Catherine Baillie Abidi, San Patten, Stephanie A Houle, Ken Hoffer, Kathryn Reeves, Stéphanie Bélanger, Anthony Nazarov, Samantha Wells

**Affiliations:** 1 Department of Child & Youth Study Mount Saint Vincent University Halifax, NS Canada; 2 Dallaire Institute for Children, Peace & Security Halifax, NS Canada; 3 MacDonald Franklin Operational Stress Injury Research Centre at Lawson Health Research Institute London, ON Canada; 4 Canadian Institute for Military and Veteran Health Research Kingston, ON Canada; 5 Center for Addictions and Mental Health Toronto, ON Canada

**Keywords:** moral injury, mental health, Canadian Armed Forces Veterans, children, deployments, conflicts, military culture, trauma-informed research, people-centered research, participatory action research

## Abstract

**Background:**

The mental health of military personnel has garnered increased attention over the last few decades; however, the impacts of perpetuating, observing, or failing to prevent acts that transgress deeply held moral standards, referred to as moral injuries, are less understood, particularly in relation to encounters with children during deployment. This paper describes a multiphased research protocol that centers around the lived experiences of Canadian Armed Forces (CAF) Veterans to understand how encounters with children during military deployments impact the well-being and mental health of military personnel.

**Objective:**

This study has four objectives: (1) highlight the lived experiences of CAF Veterans who encountered children during military deployments; (2) improve understanding of the nature of experiences that military personnel faced that related to observing or engaging with children during military service; (3) improve understanding of the mental health impacts of encountering children during military service; and (4) use participatory action research (PAR) to develop recommendations for improving preparation, training, and support for military personnel deployed to contexts where encounters with children are likely.

**Methods:**

The research project has 2 main phases where phase 1 includes qualitative interviews with CAF Veterans who encountered children during military deployments and phase 2 uses PAR to actively engage Canadian Veterans with lived experiences of encountering children during military deployments, as well as health professionals and researchers to identify recommendations to better address the mental health effects of these encounters.

**Results:**

As of January 26, 2024, a total of 55 participants and research partners have participated in the 2 phases of the research project. A total of 16 CAF Veterans participated in phase 1 (qualitative interviews), and 39 CAF Veterans, health professionals, and researchers participated in phase 2 (PAR). The results for phase 1 have been finalized and are accepted for publication. Data collection and analysis are ongoing for phase 2.

**Conclusions:**

Prioritizing and valuing the experiences of CAF Veterans has deepened our understanding of the intricate nature and impacts of potentially morally injurious events involving children during military deployments. Together with health professionals and researchers, the PAR approach empowers CAF Veterans to articulate important recommendations for developing and improving training and mental health support. This support is crucial not only during the deployment cycle but also throughout the military career, helping lessen the effects of moral injury among military personnel.

**International Registered Report Identifier (IRRID):**

DERR1-10.2196/57146

## Introduction

### Background

Research on the traumatic impacts of armed conflict and the resulting mental health consequences to military personnel has largely focused on experiences of being wounded, coming under fire, and witnessing comrades being killed or injured. In addition to these harms, military health research has accumulated evidence over the last decade pointing to the deleterious impacts that may be exerted by perpetuating, observing, or failing to prevent acts that transgress deeply held moral standards, referred to as moral injury [1,2]. International deployments increasingly involve exposure to complexities less common in previous conflict eras, including engagement with unmarked enemy combatants, suicide bombings, and encounters with children. Importantly, while deployment-related encounters with children are associated with mental health and other consequences in military personnel [3], and have been conceptualized as potentially morally injurious events (PMIEs; ie, events that can lead to mental health impacts due to a violation of moral beliefs or values) [4,5], the effects of such encounters remain largely understudied. For example, approximately 58% of the 33,500 Canadian military personnel deployed in North Atlantic Treaty Organization (NATO) operations in Afghanistan experienced PMIEs, with 43% of these individuals endorsing having experienced PMIEs involving women and children [6]. Despite the prevalence of such encounters, a recent systematic review revealed that no study has yet been conducted and peer-reviewed that focuses explicitly on the nature and impacts of deployment-related encounters with children [3]. Given that the use and recruitment of children in armed conflict are rising [7], and that PMIEs are associated with a host of mental health difficulties (eg, posttraumatic stress disorder [PTSD] and suicidality) among military personnel and Veterans [1,8,9], a concerted effort to understand the nature and effects of deployment-related encounters with children is necessary to advance policy, prevention, and intervention efforts aimed at supporting the mental wellness of military personnel and Veterans.

### Children and Armed Conflict

A total of 68% of children globally live in conflict-affected countries and over 460 million children, or 1 in 6, live in a conflict zone [10]. The number and nature of conflicts are rising and changing, often taking place in densely populated areas where civilians live. Consequently, armed conflict is increasingly and disproportionately impacting children, disrupting all aspects of their lives from health to education to peace and security. According to the latest Children and Armed Conflict report from the Special Representative of the United Nations Secretary General [11], over 24,000 grave violations were committed against children in 2022. Based on the increasing number of conflicts occurring, all projections point to an unprecedented number of grave violations committed against children in 2023.

Peace support operations are rapidly changing due to the increasingly complex security climate. Changes include the context of operations, the splintering of armed groups, rising intercommunal violence, and the increased engagement of children in conflict-related activities [10-12]. While the use of children in armed violence is not new, the strategic use of children in contemporary armed violence brings new dimensions to peace operations, particularly due to the emerging forms of recruitment and use of children in conflict and the rise of terrorism [12-14]. For the purpose of this research project, as per the Paris Principles [15], “child soldiers” or children recruited and used in armed violence are defined as:

any person below 18 years of age who is or who has been recruited or used by an armed force or armed group in any capacity, including but not limited to children, boys and girls, used as fighters, cooks, porters, spies or for sexual purposes. It does not only refer to a child who is taking or has taken a direct part in hostilities.

The changing security landscape, including the increase in children’s engagement, may put military personnel at greater risk for moral injury and other impacts [5]. For example, changing tactics, such as the increased use of girls in combat operations, may lead to unexpected encounters that violate previously held assumptions about the involvement of children in armed conflict. Despite these emerging dilemmas, little research has focused on the impacts of deployment-related encounters with children.

### Mental Health and Military Deployments

Military organizations remain inadequately prepared to deal with the complex psychological and operational requirements of service members deployed in conflict zones where children are recruited and used [5,16]. As such, military personnel are increasingly at risk for serious psychological sequelae due to exposure to these situations, which in many ways are representative of prevailing conceptualizations of PMIEs [4,5,16]. Consequences of PMIE exposure are serious, as research has demonstrated consistent associations with PTSD, depression, anxiety, and most concerningly suicidality [1,9]. Furthermore, studies have shown that exposure to and direct involvement in PMIEs is associated with PTSD symptom severity, even after controlling for exposure to combat (eg, receiving enemy fire and going on patrols) [17,18], showcasing that distinct qualities of deployment events can have particular impacts on mental health.

Encounters with children may have particularly devastating mental health implications for military personnel who hold deeply engrained individual, cultural, and religious understandings of children as innocent souls [19]. Violations of such views, for example, through perceived failure to protect children or witnessing children being harmed, may, therefore, elicit appraisals of the self or the world that leave a “stain” on the individual’s perceptions of their own and others’ moral worth [20], potentially contributing to the development or exacerbation of mental health problems, including moral injury [2]. While it is likely that individual beliefs and perceptions play a role in this process, it has also been suggested that morally challenging deployment experiences involving children represent violations of core binding moral foundations [5], adaptive characteristics inherent to humans that promote our evolutionary survival through adherence to values such as respect, purity or sanctity, and in-group loyalty [21]. Such values inherently involve the protection of vulnerable persons, including children. In this way, the moral quality of encounters with children on deployment may further burden military personnel who may already be at risk for several mental health challenges related to military service such as PTSD, major depression disorder, generalized anxiety disorder, and suicidality [22].

While symptomology may present similarly to PTSD, moral injury is unique in that feelings of shame, guilt, worthlessness, and changes in moral attitudes, including perceived institutional betrayal, are central to the etiology and maintenance of distress arising from PMIEs [23-29]. Several studies conducted thus far appear to corroborate the notion that PTSD and moral injury are distinct, showing, for example, that symptom patterns can be statistically differentiated [30] and that neural mechanisms can be distinguished when recalling traumatic compared with potentially morally injurious memories [20]. Despite advances in how we understand the impacts of specific deployment experiences [31], attention to deployment-related encounters with children is still glaringly missing from research. While there is accumulating evidence highlighting the detrimental impact of child soldiering on the children themselves [32-36], to date there is little evidence centered around people’s lived experiences documenting the effects of witnessing or interacting with children engaged in armed violence on the well-being and mental health of military personnel [3].

Considering the moral complexities of military deployments can vary widely from one region to the next, there is a need to better understand the multifaceted nature of encounters with children on deployment. For instance, a recent review by Ein et al [3] on encounters with children and their effects on military personnel demonstrated a wide range of experiences as well as consequences. Encountering children on deployment can elicit not only psychosocial and health consequences, but operational consequences as well, such as hesitation to engage with children recruited and used as soldiers. Importantly, no mechanisms are currently in place that seek to systematically document or evaluate the impact of deployment-related encounters with children, obscuring our understanding of their prevalence and consequences.

Action-oriented lived experience research is greatly needed to guide ways to better prevent and address the mental health effects of these encounters. Ein et al [3] observed that most policies and training related to encountering children on deployment are perceived by military personnel as inadequate if such policies and training exist at all. To our knowledge, there are no studies that have facilitated the collaboration of researchers and community members, including people with lived experience of encountering children during military deployments, in steering the development of recommendations for addressing the effects of deployment-related encounters with children.

In response to these important knowledge gaps, our research program provides foundational knowledge on the nature and the impact of encounters with children on deployment, as well as participatory action research (PAR) to inform future efforts to develop and implement effective policy, training, and intervention aimed at supporting the well-being of military personnel and Veterans.

## Methods

### Overview

As described below, our overall methodological approach involves centering the perspectives and lived experiences of Canadian Armed Forces (CAF) Veterans through two phases, that are (1) qualitative interviews to understand people’s lived experiences of the nature and the impacts of encounters with children during military deployments and (2) PAR to empower participants in articulating recommendations for changing predeployment, deployment, and postdeployment training and supports focused on mitigating the impacts of encountering children on deployment.

### Collaborative Approach

This study is a multiphase collaboration between the Dallaire Institute for Children, Peace and Security, the Centre for Addiction and Mental Health (CAMH), the MacDonald Franklin Operational Stress Injury Research Centre at Lawson Health Research Institute, Mount Saint Vincent University, the Canadian Institute for Military and Veteran Health Research (CIMVHR), as well as the Royal Military College of Canada. The project involves monthly team meetings with all partners to ensure that our rich multidisciplinary perspectives inform the design, data collection, analysis, and knowledge dissemination processes. The unique collaboration of mental health professionals, academic researchers, Veterans, and humanitarian practitioners, has enabled opportunities to explore this research from an interdisciplinary framework and to apply this research to multiple fields, including health, security studies, and children, peace, and security.

### Goals and Objectives

The overarching goal of this collaboration is to improve the collective understanding of the nature and impact of encountering children in armed conflict, and particularly children recruited and used in armed violence, on military personnel’s mental health and develop robust recommendations for preventing and addressing the effects of these encounters. Improved understanding is also needed to enhance operational effectiveness, including strengthening protections for military personnel and the children they seek to protect. This research project is the first of its kind to explore these important questions. The project team aims to identify opportunities to improve training, policy, and support to ensure military institutions and personnel are prepared to operate in complex conflict environments that increasingly include children.

The objectives of this study are, therefore, to (1) highlight the lived experiences of Canadian Veterans who encountered children during military deployments; (2) improve understanding of the nature of experiences that military personnel face related to observing or engaging with children during military service; (3) improve understanding of the mental health impacts of encountering children during military service; and (4) identify priority areas of action to improve preparation, training, and support for military personnel deployed to contexts where encounters with children are likely. This study is generating rich qualitative data on the nature and impacts of encountering children in armed conflict.

### Phase 1: Qualitative Interviews

#### Overview

Phase 1 of this research involved eliciting the perspectives and lived experiences of CAF Veterans through in-depth qualitative, semistructured open-ended interviews. This approach was used to uncover rich insights regarding the poorly understood phenomenon of encountering children during military deployments.

#### Participants and Recruitment

The participants include CAF Veterans who had direct experiences (eg, exposed to, witnessed, or engaged) with children, particularly children recruited and used in armed violence, during military deployments or operations. Participants had to be 18 years of age or older and live in Canada. The team aimed to interview about 20 participants, of whom at least 10 were women, and at least 6 of whom were Francophone.

Participants were recruited using advertisements outlining the study and providing study contact information, which were circulated among stakeholder institutions in Canada for distribution to their membership (eg, Veteran Trainers to Eradicate the Use of Child Soldiers, Dallaire Institute for Children, Peace, and Security, and the CIMVHR). Additionally, we distributed the advertisement on social media (eg, Facebook [Meta Platforms, Inc] and Twitter [Twitter Inc]). English and French versions of the advertisement were distributed. English advertisements instructed prospective participants to contact the project coordinator by telephone or email. The Project coordinator confirmed eligibility and provided an overview of the study. Additionally, permission to contact the participant through email and Webex (Cisco), the secure network used by CAMH, was obtained at this point. For those who elected to take part in the study, the project coordinator scheduled an interview. The French language advertisement directed individuals interested in participating to contact the French interviewer, who conducted the screening and consent procedures described above and conducted the interview in French.

#### Procedure

Research team members from the MacDonald Franklin Operational Stress Injury Research Centre and CAMH conducted the interviews due to their trauma-informed and counseling expertise, qualitative research experience, or their extensive knowledge and experience related to children and armed conflict. A trauma-informed approach was central to the interview process and included strategies such as collecting participant contact information and emergency contact information from participants prior to the interview, well-being checks throughout the interview process, prior arrangements with mental health providers in advance of the interviews to ensure availability of support if required, and provision of a list of available mental health resources for the participants.

Before the interview, the participant’s consent was obtained using Webex and an eConsent platform provided using Research Electronic Data Capture (REDCap). After the interview, participants were asked demographic questions. The responses to these demographic questions were entered by the interviewer directly into REDCap and were linked to individual interview data using a unique identification number assigned to the participant. Aggregate questionnaire responses are used only for descriptive information about the sample.

During the semistructured interviews, participants were asked about the nature of experiences they had faced related to observing or engaging with children during their military service. Aligning with the most current understanding of the domains of the impact of moral injury, participants were also asked about the impact of these experiences with domains of exploration, including (1) alterations in self-perception (eg, changes in identity and confusion about whether there is a moral code); (2) psychological impact of alterations in moral thinking (eg, changes in moralism, appraisal of others, and rumination); (3) relational impacts (eg, lack of interest or investment in social relationships, change in interactions with children, loss of belonging, and social isolation); (4) emotional aftermath (eg, shame, guilt, and anger); and (5) spirituality (eg, loss of life purpose and loss of spiritual or religious beliefs).

We also aimed to explore whether these experiences and the effects of these experiences differed for men and women, as well as what kinds of training and support they felt were needed for military personnel who may be deployed to regions where child soldiers operate.

Interviews lasted approximately 60 minutes. Participants were provided a CAD $50 (US $36.60) gift card as remuneration, and the gift card was delivered to the participant by the project coordinator by email or mail, per the participant’s wishes.

Interviews were audio-recorded using Webex’s recording feature. The interviewer took field notes during the interview to elaborate on the content of the interview, such as nonverbal cues. Raw audio files and transcripts are stored on the CAMH server, separately from other documents containing personally identifiable information, to prevent identifying participants. All data were deidentified, with numeric identifiers used in place of names. Pseudonyms were used in place of names, where applicable. Upon completion of the interviews, all materials were transferred to the project coordinator, and the interviewers’ access to the audio files and the REDCap eConsent forms was removed. For participants who did not wish to be contacted in the future, emails and contact information were destroyed by the project coordinator once all interviews were completed. Contact information for all other participants is saved on the CAMH server.

#### Analyses

Transcribed interviews were analyzed using Braun and Clarke’s [37] 6-stage approach to thematic analysis to identify themes within the interview data. The process involves familiarization and initial coding, organizing codes into potential themes, summarizing data relevant to each theme, reviewing, verifying, defining, and then naming each theme. NVivo software (version 12; Lumivero, 2017) was used to manage the data. Interviews were coded by several authors independently, including the interviewers and members of the research team. The research team met regularly to review coded excerpts and to resolve any discrepancies through group consensus.

Coding was done in an ongoing manner as interviews occurred. A codebook was created to capture evident themes. As new themes were identified throughout the study, the codebook was refined, and previously coded interviews were reanalyzed using the updated codebook. This iterative approach allowed the subsequent interviews to be tailored around unanticipated themes identified during analysis. This ensured that the lived experiences of the participants were captured and validated appropriately. Member-checking was done where coders were unsure of or needed further clarification on themes. Upon completion of the analysis, a qualitative synthesis was developed to describe in detail the thematic contents of the interview data.

### Phase 2: PAR

#### Overview

The second phase of the research involved PAR. PAR is a qualitative research methodology that actively engages the community in all aspects of the research, incorporating the perspectives and lived experiences of participants in meaningful ways [38,39]. It is considered a category of action research [40], which involves the systematic collection and analysis of information to identify real and salient issues, act on those issues, and make social change. In PAR, researchers and community members collaborate to explore a problem and identify priorities for action through a cyclical and responsive process of research, reflection, action, evaluation, and modification [41]. Using this research-to-action approach, study participants, who are people with lived experience of encountering children during military deployments, particularly children recruited and used in armed violence, steer the development of recommendations, rather than being passive research subjects.

**Figure 1:**
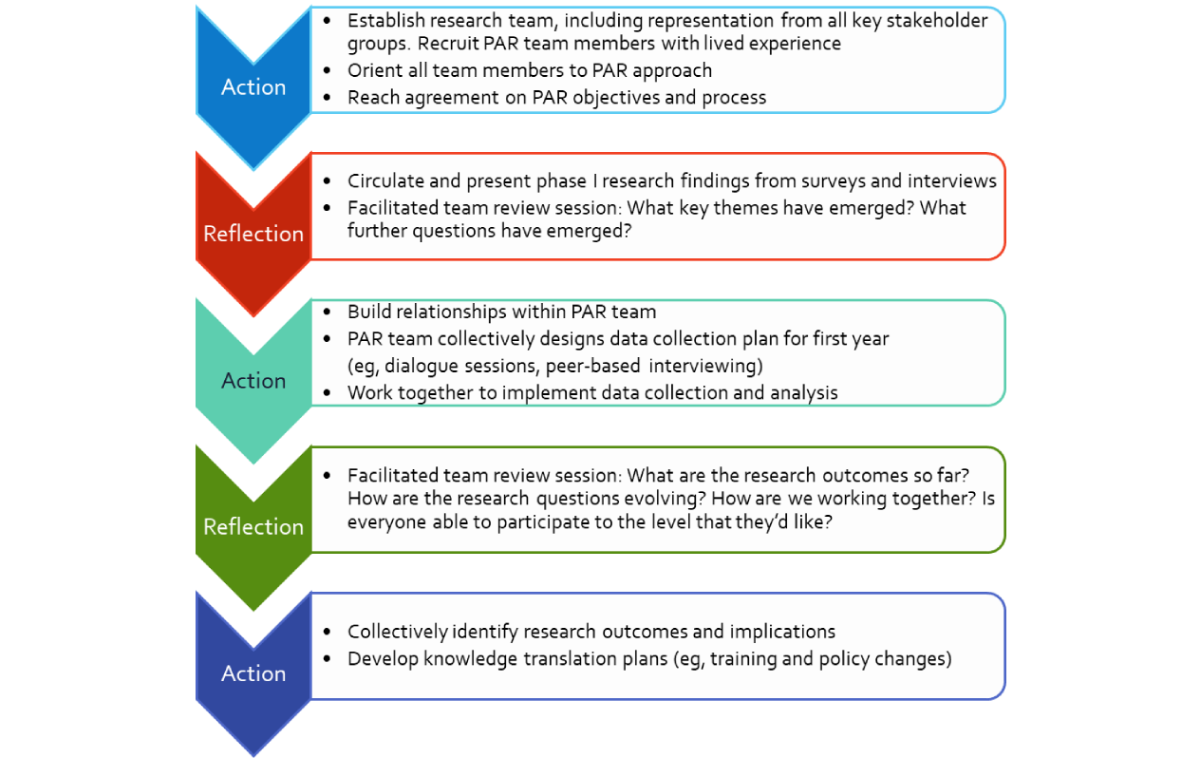
PAR project stages. PAR: participatory action research.

The PAR approach involves iterative cycles of reflection, data collection, and action. Each phase of the PAR project organically builds on previous cycles, with strong involvement of, and direction from, an interdisciplinary team of key stakeholders. As working relationships develop within the research team, information and perspectives are shared, questions emerge, plans are formed for how best to address those questions, more data are gathered, the team collaborates in analyzing and interpreting the data, and more questions emerge. Figure 1 summarizes the iterative process by which the PAR project is unfolding.

#### Participants and Recruitment

Phase 2 of the research project includes CAF Veterans as well as health professionals and researchers from varied disciplines including health, peace and security, and child and youth study. The aim of the initial phase of the PAR project was to recruit approximately 5-10 Veterans, including at least 2 women. Participants could be located anywhere in Canada, speak either official language, and are military Veterans who have self-identified lived experiences with trauma or moral injury and encountered children during deployment, particularly children recruited and used in armed violence.

**Figure 2:**
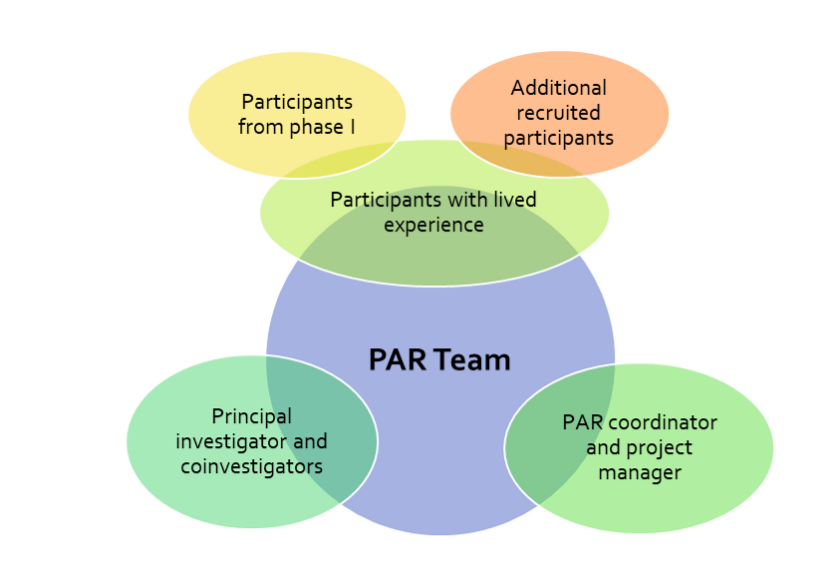
PAR team composition. PAR: participatory action research.

Participants were recruited through posters distributed among stakeholder institutions in Canada (eg, Veteran Trainers to Eradicate the Use of Child Soldiers, Dallaire Institute for Children, Peace and Security, and the CIMVHR) for distribution to their membership, including through social media (eg, Facebook, Twitter, and Instagram [Meta Platforms]). The posters instructed interested individuals to contact the PAR coordinator by email or telephone. The research team also followed up with participants from phase 1 who agreed to be contacted for future phases of the study, as per the previous protocol approved by Dalhousie (#2021-5723 and #2022-6163), CAMH (#2021-045 and #2022-078), Mount Saint Vincent (#2022-015 and #2022-048), Lawson (#11430 and #12699), and Western (#120351 and #121499) Research Ethics Boards. Please see Figure 2 below for an explanation of the PAR team composition.

#### PAR Process

##### Overview

The first stage of the PAR project involved the collaborative interpretation of initial phase 1 findings with the full PAR team, including people with lived experience. The PAR team has so far engaged in a series of 6 participatory analysis workshops that involved sharing findings from the phase 1 interviews and information created in previous workshops through an iterative process of questioning, data generation, and interpretation. In this first stage of the PAR project, the PAR team reviewed phase 1 findings. Note that the PAR research team was presented with general findings from phase 1 that had been anonymized and compiled thematically. Next, they engaged in a discussion to answer interpretive questions that are, (1) how do the phase 1 study findings resonate (or not) with your own perspective, expertise, or experience encountering children during deployment?; (2) what questions do the phase 1 findings leave for us to further explore in relation to moral injury?; (3) who should we engage in addressing these questions, and how do we engage them in ways that feel safe and productive?; and (4) what are the implications of these findings in terms of providing preparatory training and support services for military personnel?

The PAR project’s data collection does not involve traditional research instruments, but rather the interpretive discussion emerged through a series of participatory analysis workshops (the first workshop agenda is detailed in Multimedia Appendix 1). At the time of writing, the PAR process has unfolded over a series of 6 workshops that included CAF Veterans, researchers, and health service providers. These workshops were spread over 16 months and were 2 hours to 2 days in duration.

The first workshop was in October 2022 and the most recent sixth workshop was held in January 2024. Additional workshops continue to be organized based on the guidance and recommendations of the Veteran participants. Below is a synopsis of the focus for each workshop so far.

- Workshop 1 (October 2022, in-person): introductions, share overall objectives or goals, share key findings from phase 1, discuss implications of findings in terms of providing preparatory training and support services for military personnel who have encountered children in combat situations, and identify other stakeholders who should be engaged in these services
- Workshop 2 (January 2023): Review of workshop 1 findings, gap analysis of predeployment training for CAF personnel, gap analysis of policies to guide CAF personnel in how to prepare for encounters with children during deployment, and gap analysis of supports for CAF personnel after they have experienced encounters with children during deployment
- Workshop 3 (February 2023): Review of workshop 2 findings; discussion of the health effects of encountering children during deployments to inform policy, practice, and supports; identify circles of support pre-, during, and postdeployment; and discussion of best practices for support services
- Workshop 4 (March 2023): Review of workshop 3 findings, identify key messages and recommendations emerging from phases 1 and 2, and knowledge dissemination ideas
- Workshop 5 (July 2023, in-person): review of workshop 4 findings, discussion of concepts of childhood and military culture from the perspectives of Veterans and health professionals, and explore strategies for the improvement of care relating to moral injury, particularly in relation to the health effects of encountering children during deployment
- Workshop 6 (January 2024, in-person): Review of findings from phases 1 and 2 with only women PAR team members to explore findings and propose possible actions from a gender-responsive perspective

In PAR processes, the data collection and data analysis are integrated into each exercise, rather than as separate steps. The collaborative interpretation process occurs within each participatory analysis workshop using techniques such as (1) free list and pile sort of key messages emerging from phase 1 to categorize and prioritize emergent themes; (2) stakeholder identification and mapping of stakeholders’ level of influence and degree to which they are affected; (3) arts-based elicitation of themes and implications; and (4) mapping implications for prevention, preparation, and intervention, based on criteria of impact and feasibility.

In keeping with the PAR methodology, all findings are shared with the participants to seek their feedback and input on the accuracy and appropriateness of representation. This process involves a series of collaborative editing and adjusting to ensure the whole PAR team is satisfied with the final themes and knowledge products. A lay summary of the PAR study results with no identifying information will be prepared and made publicly available.

### Ethical Considerations

For both research phases, ethical approval was obtained from each primary collaborating site, including Dalhousie University (for the Dallaire Institute for Children, Peace, and Security); the CAMH; the University of Western Ontario (for the Lawson Health Research Institute and the MacDonald Franklin Operational Stress Injury Research Centre); and Mount Saint Vincent University (approval numbers are listed in the *Participants and Recruitment* subsection of the *Phase 2: PAR* section). Strict protocols are in place for sharing data between institutions. Cross-site data sharing agreements were drawn up outlining protocols for data governance and data sharing, which all parties are adhering to closely.

### Phase 1

Strict adherence to institutional ethical standards and protocols is being maintained. For one-to-one interviews in phase 1, we used Webex, a secure videoconferencing software. Participants were informed of best practices for maintaining privacy and confidentiality in research settings and were notified of the additional risks associated with research using online platforms (ie, email and Webex). Before beginning the research session, the interviewer ensured the participant’s space was private to avoid disruption and breaches of confidentiality. Participants were advised that the session would be audio-recorded. A link to the Webex session was provided to the participant through email. Only invited participants who signed the consent form were permitted to access the meeting.

A trauma-informed approach was used throughout the interview process due to the sensitive and potentially distressing nature of the subject matter discussed during interviews. Before the interview, participants were provided contact information for emergency clinical mental health support and other support and information. At the start of the session, the interviewer obtained an emergency phone number, a secondary phone number, and the participant’s address in case the need arose to confirm the safety of the participant. And finally, during the interview, the interviewer assessed the safety of the participant on an ongoing basis, and if deemed necessary, was prepared to terminate the interview early and provide options for available mental health support to the participant. Having clinically trained interviewers was a significant asset to this project, allowing both interviewees and other research team members to feel comfortable that the well-being of participants was being prioritized.

To address the potential for vicarious trauma that may be incurred by the interviewer, the research team held debrief sessions with the interviewer and offered space and time for the interviewer to reflect on the interview experience. A trained psychologist who is a member of the team was also available in the event of any psychological distress exhibited by the interviewer.

### Phase 2

Participants with lived experience were provided with a copy of a research concept form (RCF) through email by the PAR coordinator. The contents of the RCF were discussed with the PAR team members after they had time to review the RCF, including a review of the purpose and nature of the project, privacy, confidentiality, the participant’s rights, and the right to withdraw from the study at any time without giving a reason. Participants were reassured that whether they participate or complete the full study will not affect any treatment needs that they might have at the CAMH, or hospitals affiliated with the Lawson Health Research Institute, now or in the future. Participants were given the opportunity to ask questions about the project and after the consent discussion, prospective participants were sent a link to the eConsent. Participants were told that some data (eg, collected anonymously or as part of a group discussion) may not be identified as belonging to the participant and cannot be destroyed.

Before the first workshop, all PAR members were asked to review and sign an ethics commitment form focusing on maintaining the confidentiality of the group members (Multimedia Appendix 2). Additionally, during our first workshop, participants were invited to develop community norms, that is, the group’s agreements regarding confidentiality, respectful, and inclusive discussion. As in focus group research, participants are asked to respect the privacy and confidence of all other participants and agree not to disclose any personal information shared during the meetings or workshops. These expectations are also outlined in the RCF. Names of people with lived experience will not appear in any publications or reports about this research unless approved by the participants.

Finally, all participants were provided with a list of services in the event they needed information or support related to mental health and trauma. For their safety, participants were asked for an emergency contact number, alternate phone number, and address before they participated in virtual or in-person PAR activities. Participants were advised that they could opt out of any PAR activities should they feel uncomfortable, and that if the research team is concerned for their immediate safety, the research team may contact them, their emergency contact, or emergency responders to follow-up. Importantly, our PAR team included a trained psychologist, who was present during all data collection and analysis and is available to discreetly offer support during or after any signs of distress among the PAR team.

### Special Consideration for Military Personnel: Phases 1 and 2

All Veteran participants were advised not to divulge operationally sensitive information during their interviews or PAR workshops to avoid contravention of the Security of Information Act. Participants were instructed to relate only the types of encounters with children and the impact they had on them. All participants were reminded not to disclose information related to operational details, including (1) identity of key national or allied commanders, leaders, units, interpreters, insurgents, other special operations entities; (2) dates, time, locations of special operations; (3) linking code words and operations to high-level security clearance access; (4) targeting Boards—decision processes or parameters; (5) specific rules of engagement; (6) intelligence gathering methodology and sources; (7) weapon and sensor capabilities and limitations of any platform (land, sea, and air); (8) manning and employment of special operations units; (9) operational and tactical plans and procedures; (9) personal identity of enemy killed in action or taken prisoner of war; (10) covert activities and collection methods to gain intelligence and operational advantage; (11) identify encryption and cryptographic systems—vulnerabilities or limitations; (12) electronic warfare capabilities and signal analysis; (13) research and technical development initiatives regarding future operations; (14) platform capabilities and employment—numbers, manning, and operational limitations; and (15) Government of Canada or CAF Strategic Defence and Security Plans.

If information was inadvertently mentioned during the interview, the interviewer was instructed to redact that information from the audio recording before it was saved to the CAMH server or recorded during the PAR group discussions. Fortunately, all participants have abided by these instructions. To date, no participant has made an inadvertent statement that would compromise national security.

### Timeline

Research Ethics Board approval for phase 1 was secured in June 2021 and participant recruitment and data collection occurred between November 2021 and June 2023. Findings from phase 1 are expected to be published in 2024. Research Ethics Board approval for phase 2 was secured in July 2022 and continues to evolve. Participant recruitment and data collection for phase 2 began in the fall of 2022 and remains ongoing.

## Results

As of January 26, 2024, a total of 55 participants and research partners have participated in the 2 phases of the research project. A total of 16 Canadian Veterans participated in phase 1 (qualitative interviews) and 39 Canadian Veterans, health professionals, and researchers participated in phase 2 (PAR). The research results stemming from phases 1 and 2 point to the complexities and significance of the impacts of encountering children during military deployments. Six key themes have been identified that include, (1) the types of encounters with children during deployment vary and include experiences of heightened ambiguity with children in conflict contexts; (2) the contextual factors, including the mission framework, the deployed environment, and personal contexts, shape the impact of encounters; (3) how military personnel appraise encounters with children influences the impact; (4) the impacts of encounters with children vary in intensity and scope and are part of a larger stressor of deployment; (5) various coping strategies are used to manage such impacts during and after deployments; and (6) formal and informal support following encounters with children is likely to mitigate mental health impacts. The preliminary findings also point to the possibilities for enhancing preparatory training, care, and support at all phases of military deployment for those who face these potentially morally injurious experiences. The results for phase 1 have been finalized and are accepted for publication. Data collection and analysis are ongoing for phase 2.

## Discussion

Prioritizing and valuing the experiences of CAF Veterans has resulted in an increased understanding of the complexities of the nature and impacts of PMIEs involving children during military deployments. The qualitative study with Canadian Veterans (phase 1) generated new knowledge in relation to the interconnections between military mental health, deployment experiences, and observing or engaging with children during military service, an overlooked area within military mental health research. Additionally, the PAR approach (phase 2) empowered Canadian Veterans to articulate important recommendations for changing predeployment, deployment, and postdeployment training and support to reduce moral injury among military personnel. While the overall analysis of the 2 phases is ongoing, we acknowledge that expanding the number and diverse identities of the participants, particularly in relation to gender and race, will be an important future research plan to ensure an intersectional analysis is applied to the complexities of the impacts of encountering children during international deployments. We encourage researchers to conduct similar research within other peace support–contributing states to continue building our collective knowledge of the connections between PMIEs, such as encountering children, and mental health to inform better prevention and intervention programming.

From a research design perspective, this novel, multilayered, qualitative, trauma-informed, Veteran-centered, and action-oriented project has resulted in key learnings related to the development of research protocols, implementation, analysis, and knowledge dissemination in Veteran mental health. Human-centered and trauma-informed principles guided the design of both phases of the study and were strengthened by a collective commitment among the research team to prioritize care and respect within our learning environment. The trauma-informed design and multidisciplinary team were key strengths contributing to the creation of meaningful, respectful, and care-centered research processes that prioritized the well-being of participants and the research team. The iterative process of collecting and analyzing in-depth qualitative data (phase 1), then validating and deepening the analysis through PAR methods (phase 2), has produced a robust set of findings, as well as recommendations for real-world policy and program implications. The participants expressed appreciation for and positive benefits from their engagement in the PAR processes, building a sense of solidarity, productivity, and empowerment. Moreover, the colearning and participatory action approach taken with multidisciplinary team members, including Canadian Veterans, served to propel key knowledge mobilization activities to address mental health challenges among military personnel and substantiate future research directions to be investigated from the perspectives of our various disciplines, highlighting the impact that integrative teams can produce.

## Appendix

Multimedia Appendix 1 Participatory action research workshop 1 agenda.

Multimedia Appendix 2 Ethics commitment form.

## Abbreviations

CAF: Canadian Armed Forces
CAMH: Centre for Addiction and Mental Health
CIMVHR: Canadian Institute for Military and Veteran Health Research
NATO: North Atlantic Treaty Organization
PAR: participatory action research
PMIE: potentially morally injurious event
PTSD: posttraumatic stress disorder
RCF: research concept form
REDCap: Research Electronic Data Capture

## Data Availability

The data sets generated and analyzed during this study are not publicly available due to the sensitive nature of this research and the ethical commitments to study participants.
