# Supplementary figures and images for: Canadian Armed Forces Veterans’ Perspectives on the Effects of Exposure to Children in Armed Conflict During Military Service: Protocol for a Qualitative Study
Source: JMIR Res Protoc. 2024 Jun 14;13:e57146. doi: 10.2196/57146 (PMC11214030; doi:10.2196/57146)

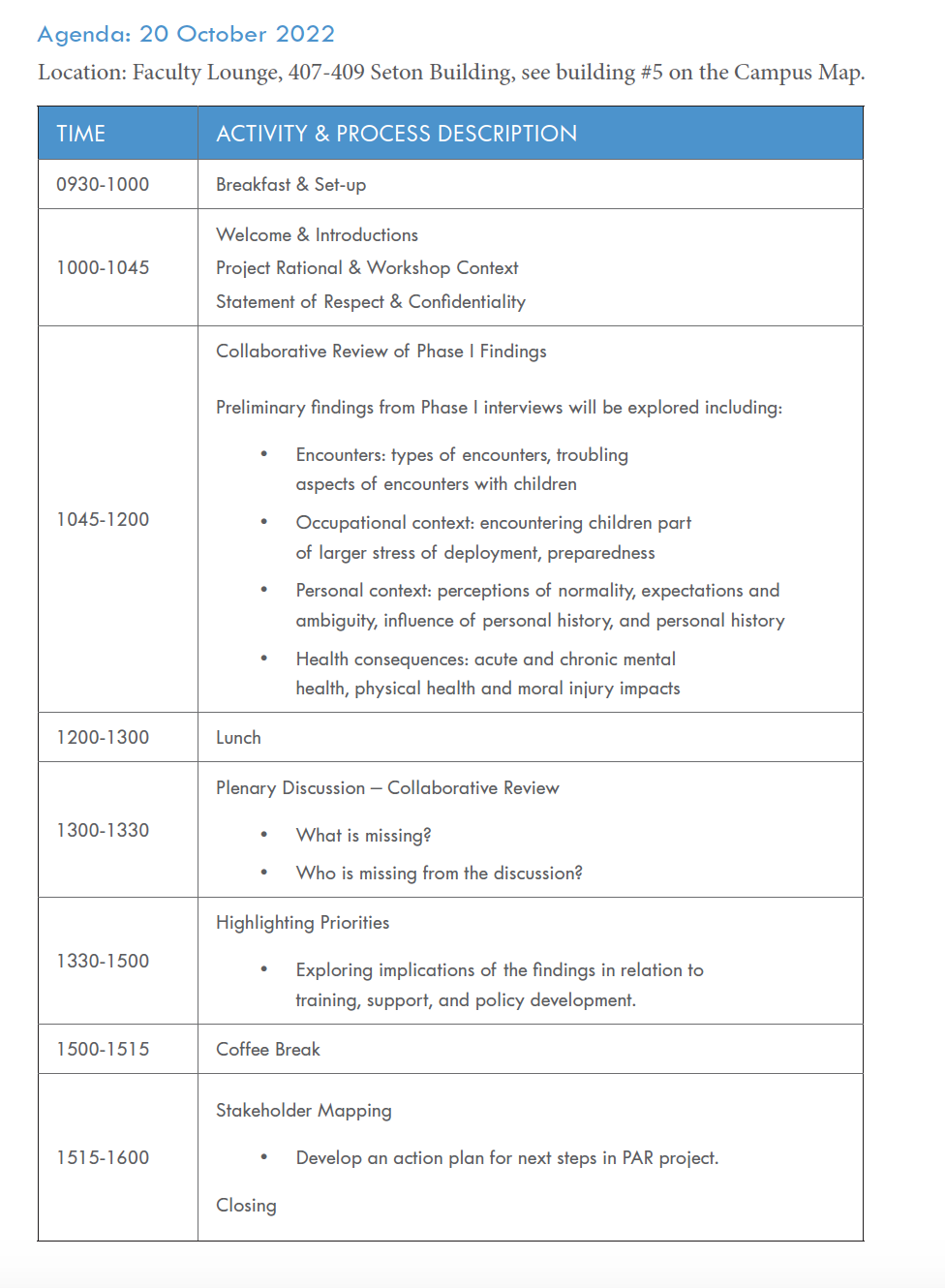

Supplement: Multimedia Appendix 1 [file resprot_v13i1e57146_app1.docx]

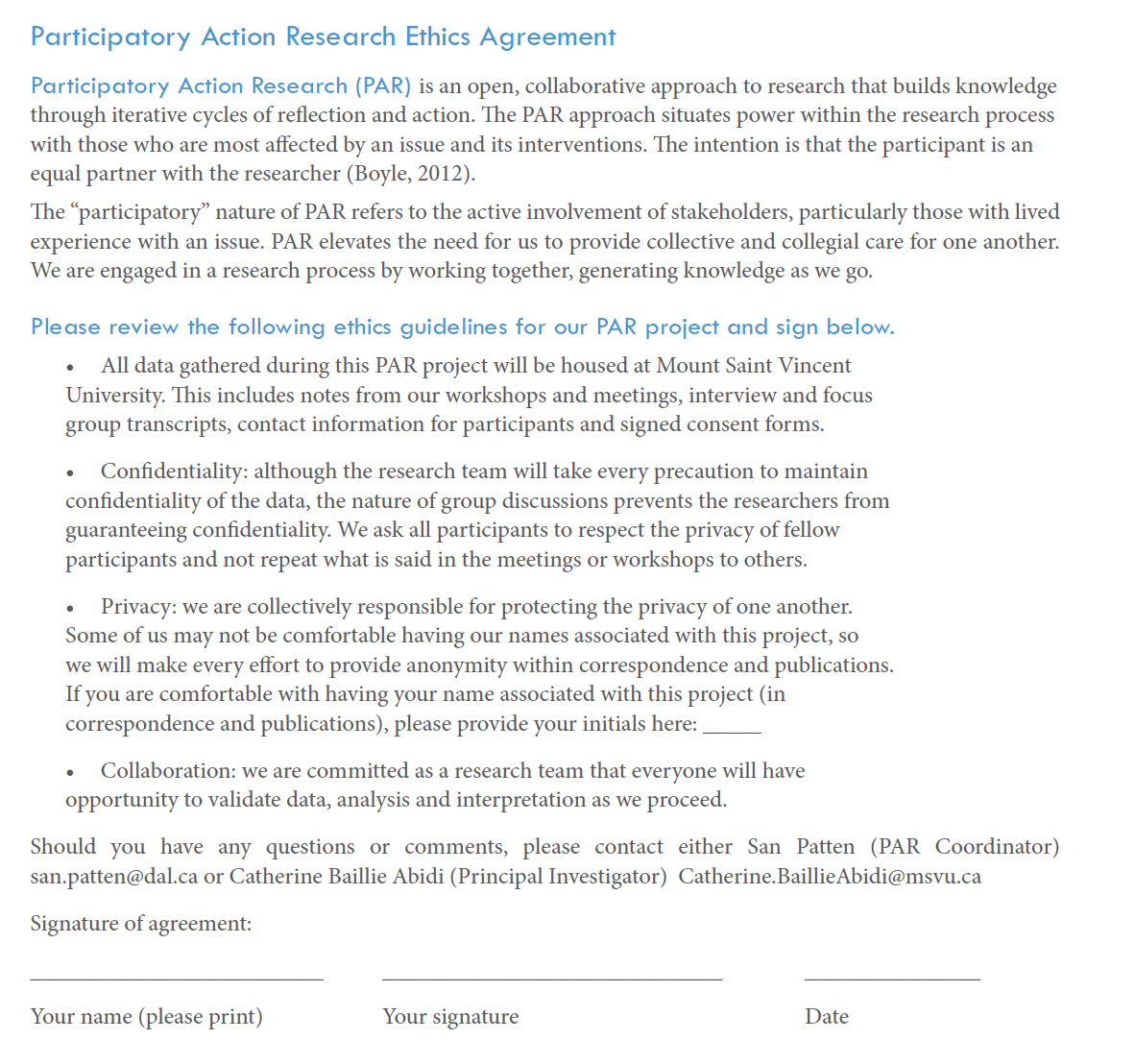

Supplement: Multimedia Appendix 2 [file resprot_v13i1e57146_app2.docx]
